# Supplementary material for: Targeting dependency on a paralog pair of CBP/p300 against de-repression of KREMEN2 in SMARCB1-deficient cancers
Source: Nat Commun. 2024 Jun 5;15:4770. doi: 10.1038/s41467-024-49063-w (PMC11153594; doi:10.1038/s41467-024-49063-w)
Supplement: Supplementary file 1 — Supplementary Information [file 41467_2024_49063_MOESM1_ESM.pdf]

Supplementary Information

Title

Targeting Dependency on a Paralog Pair of CBP/p300 against De-repression of KREMEN2 in SMARCB1-Deficient Cancers

Authors

Mariko Sasaki[1], Daiki Kato[2], Karin Murakami[2], Hiroshi Yoshida[3], Shohei Takase[1], Tsuguteru Otsubo[2], Hideaki Ogiwara[1*]

Affiliations

[1] Division of Cancer Therapeutics, National Cancer Center Research Institute, 5-1-1, Tsukiji, Chuo-ku, Tokyo 104-0045, Japan.
[2] Cancer Research Unit, Sumitomo Pharma Co., Ltd, 3-1-98 Kasugade-naka, Konohana-ku, Osaka 554-0022, Japan.
[3] Department of Diagnostic Pathology, National Cancer Center Hospital, 5-1-1, Tsukiji, Chuo-ku, Tokyo 104-0045, Japan.

Supplementary Figure 1

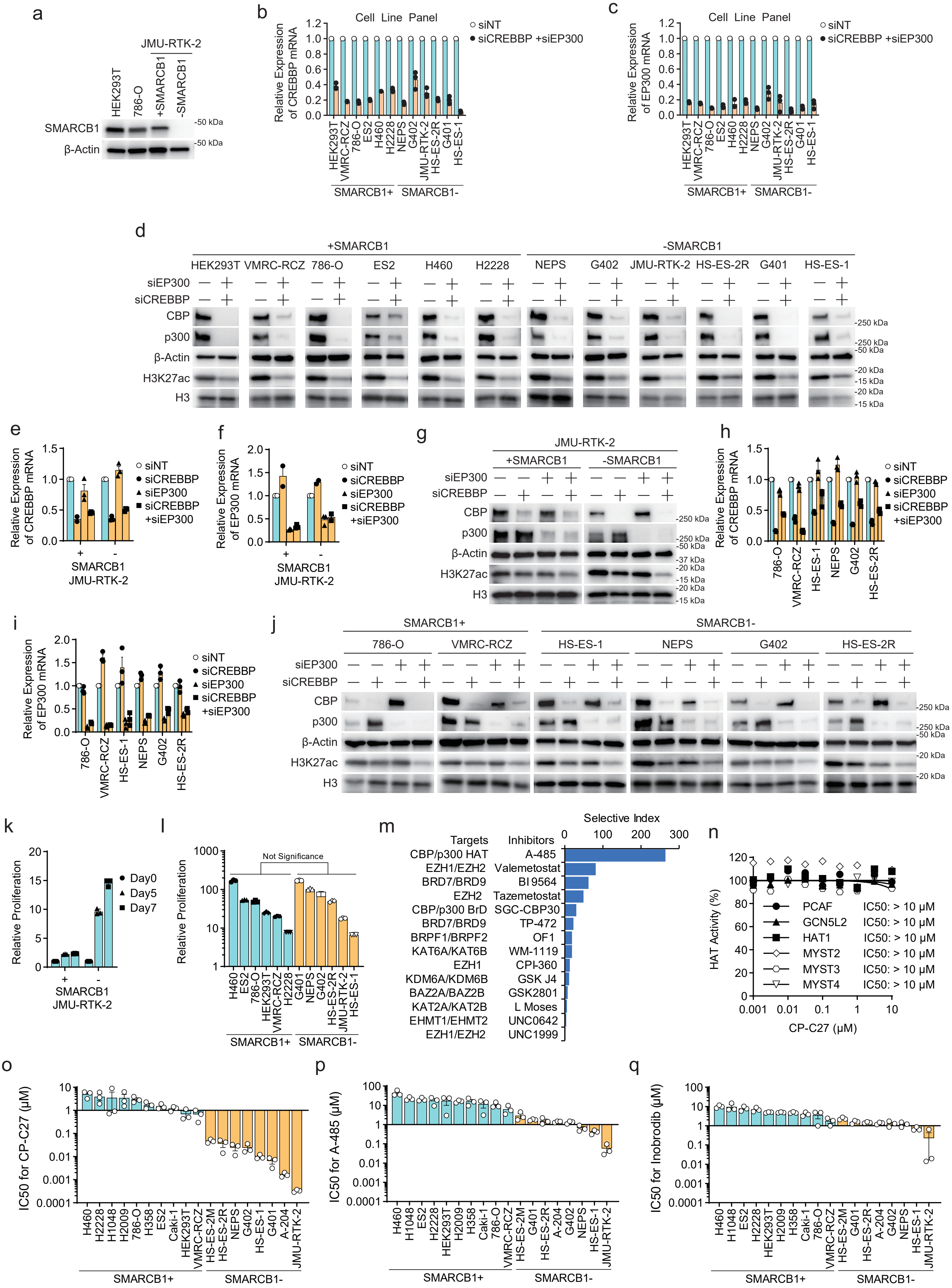

Supplementary Figure 1. Simultaneous inhibition of CBP/p300 causes synthetic lethality in SMARCB1-deficient cancer cells.

a, Immunoblot analysis of SMARCB1 and β-actin expression in SMARCB1-proficient cells (HEK293T and 786-O), JMU-RTK-2 +SMARCB1 cells, and JMU-RTK-2 -SMARCB1 cells.

b, c, Relative expression of CREBBP (b) and EP300 (c) mRNA in SMARCB1-proficient (HEK293T, VMRC-RCZ, 786-O, ES2, H460, and H2228) and SMARACB1-deficient (NEPS, G402, JMU-RTK-2, HS-ES-2R, G401, and HS-ES-1) cell lines transfected for 48 h with siRNAs specific for CREBBP+EP300, or with NT (non-targeting) siRNA. Data are presented as the mean ±SD (standard deviation), n = 3 independent experiments.

d, Immunoblot analysis of CBP, p300, H3K27ac, H3, and β-actin expression in SMARCB1-proficient (HEK293T, VMRC-RCZ, 786-O, ES2, H460, and H2228) and SMARACB1-deficient (NEPS, G402, JMU-RTK-2, HS-ES-2R, G401, and HS-ES-1) cell lines transfected for 48 h with siRNAs specific for CREBBP+EP300, or with NT siRNA.

e, f, Relative expression of CREBBP (e) and EP300 (f) mRNA in JMU-RTK-2 +SMARCB1 cells and JMU-RTK-2 -SMARCB1 cells transfected for 48 h with siRNAs specific for CREBBP and/or EP300, or with NT siRNA. Data are presented as the mean ± SD, n = 3 independent experiments.

g, Immunoblot analysis of CBP, p300, H3K27ac, H3, and β-actin expression in JMU-RTK-2 +SMARCB1 cells and JMU-RTK-2 -SMARCB1 cells transfected for 48 h with siRNAs specific for CREBBP and/or EP300, or with NT siRNA.

h, i, Relative expression of CREBBP (h) and EP300 (i) mRNA in SMARCB1-proficient (786-O and VMRC-RCZ) and SMARACB1-deficient (HS-ES-1, NEPS, G042, and HS-ES-2R) cell lines transfected for 48 h with siRNAs specific for CREBBP and/or EP300, or with siNT RNA. Data are presented as the mean ±SD, n = 3 independent experiments.

j, Immunoblot analysis of p300, H3K27ac, H3, and β-actin expression in SMARCB1-proficient (786-O and VMRC-RCZ) and SMARACB1-deficient (HS-ES-1, NEPS, G042, and HS-ES-2R) cell lines transfected for 48 h with siRNAs specific for CREBBP and/or EP300, or with NT siRNA.

k, Proliferation rate of JMU-RTK-2 +SMARCB1 and JMU-RTK-2 -SMARCB1 cells. Cells were seeded (Day 0) and then incubated for 7 days (Day 7). The proliferation rate is calculated as the cell viability on Day 7 relative to that on Day 0. Data are presented as the mean ± SD, n = 3 independent experiments.

l, Proliferation rate of SMARCB1-proficient (H460, ES2, 786-O, HEK293T, and H2228) and SMARACB1-deficient (G401, NEPS, G402, HS-ES-2R, JMU-RTK-2, and HS-ES-2R). Cells were seeded (Day 0) and then incubated for 7 days (Day 7). The proliferation rate is calculated as ratio of cell viability on Day 7 relative to that on Day 0. Data are presented as the mean ± SD, n = 3 independent experiments.

m. Selectivity Indexes for JMU-RTK-2 -SMARCB1 cells treated with the indicated inhibitors. Cells were treated with the indicated inhibitors for 6 days and IC50 (50% inhibition concentration) values were calculated based on cell viability. The Selectivity Index is calculated as the ratio of the 50% inhibitory concentration (IC50) of JMU-RTK-2 +SMARCB1 cells relative to that of JMU-RTK-2 -SMARCB1 cells.

n, Histone acetylation (HAT) activity of PCAF, GCN5L2, HAT1, MYST2, MYST3, and MYST4 in vitro. The IC50 values denote the inhibitory effects of CP-C27.

o, p, q, IC50 values for CBP/p300 inhibitors CP-C27 (o), A-485 (p), and inobrodib (q) in SMARCB1-proficient (H460, H1048, H2009, H2228, 786-O, H358, Caki-1, HEK293T, VMRC-RCZ, and ES2) and SMARCB1-deficient (HS-ES-2M, HS-ES-2R, A-204, NEPS, G401, G402, HS-ES-1, and JMU-RTK-2) cell lines. Cells were treated with inhibitors for 6 days and IC50 values were calculated based on cell viability. Data are presented as the mean ± SEM (standard error of the mean), n = 3 independent experiments.

Supplementary Figure 2

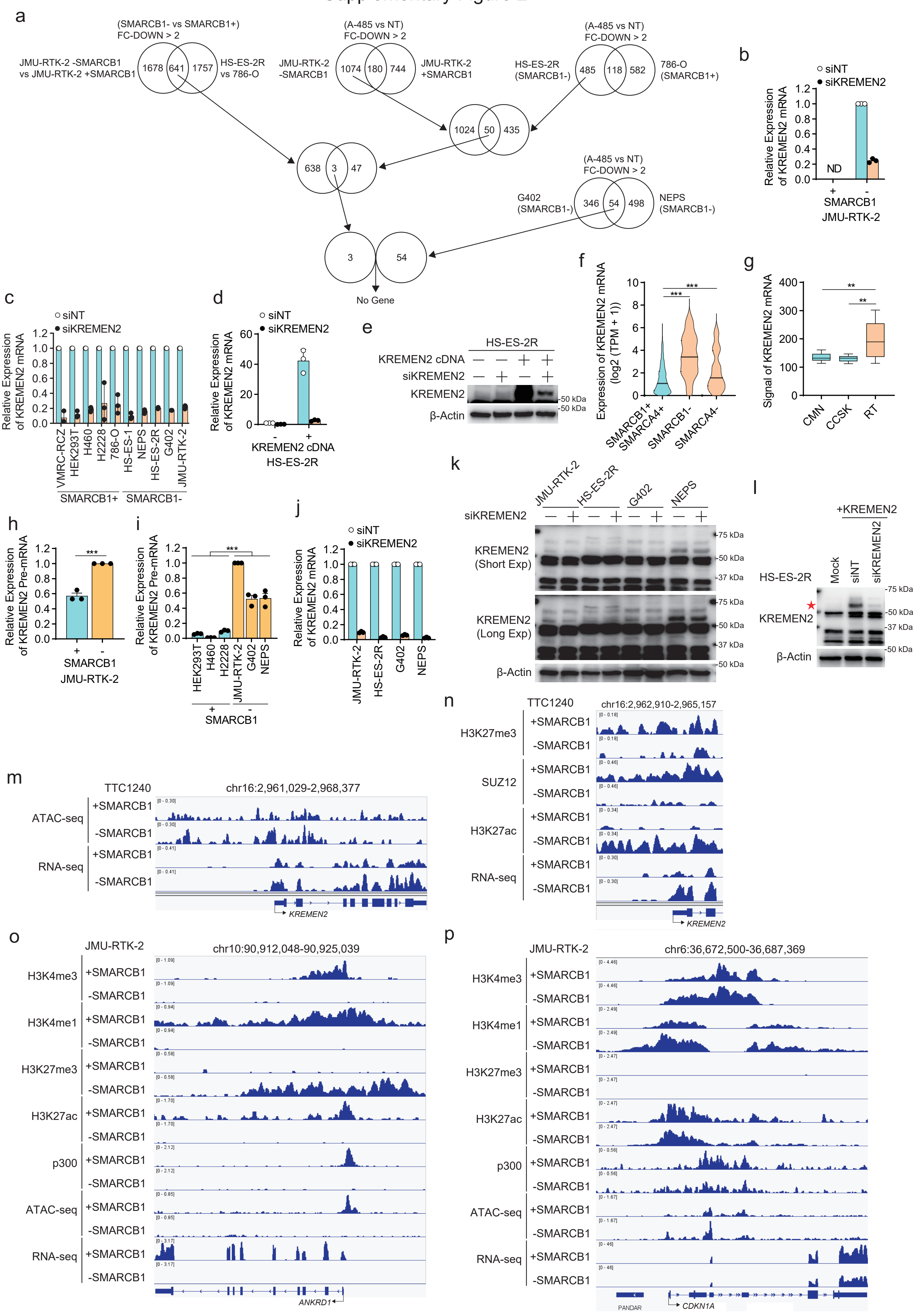

Supplementary Figure 2. SMARCB1 deficiency upregulates expression of the KREMEN2 gene.
a, Schematic flow illustrating the method used to identify determinants of synthetic lethality in SMARCB1-deficient cells treated with a CBP/p300 inhibitor. None was identified and selected as a gene that is downregulated in SMARCB1-deficient cells, and downregulated in A-485 treated SMARCB1-deficient cells but not SMARCB1-proficient cells. A set of 641 genes that were concordantly downregulated in SMARCB1-deficient cells (JMU-RTK-2, HS-ES-2R), but not in SMARCB1-proficient cells (JMU-RTK-2 +SMARCB1, 786-O), and a set of 50 genes that were concordantly downregulated in SMARCB1-deficient cells (JMU-RTK-2, HS-ES-2R) treated with A-485, but not in SMARCB1-proficient cells (JMU-RTK-2 +SMARCB1, 786-O), was isolated. Then, a set of three genes overlapping these gene sets was isolated. In addition, a set of 54 genes that were concordantly downregulated in two other SMARCB1-deficient cells (G402, NEPS) treated with A-485 was identified. No genes overlapping the two gene sets were identified.
b, Expression of KREMEN2 mRNA (relative to that in siNT-transfected JMU-RTK-2 -SMARCB1 cells) in JMU-RTK-2 +SMARCB1 and JMU-RTK-2 -SMARCB1 cells transfected for 48 h with the indicated siRNAs. KREMEN2 mRNA was not detected (ND) in JMU-RTK-2 +SMARCB1 cells. Data are presented as the mean ± SD (standard deviation), n = 3 independent experiments.
c, Expression of KREMEN2 mRNA (relative to that in siNT-transfected cells) in SMARCB1-proficient (VMRC-RCZ, HEK293T, H460, H2228, and 786-O) and SMARCB1-deficient (HS-ES-1, NEPS, HS-ES-2R, G402, and JMU-RTK-2) cell lines transfected for 48 h with the indicated siRNAs. Data are presented as the mean ± SD, n = 3 independent experiments.
d, Expression of KREMEN2 mRNA (relative to that in siNT-transfected HS-ES-2R mock cells) in HS-ES-2R mock and HS-ES-2R +KREMEN2 cells transfected for 48 h with the indicated siRNAs. Data are presented as the mean ±SD, n = 3 independent experiments.
e, Immunoblot analysis of KREMEN2 and β-actin expression in HS-ES-2R mock and HS-ES-2R +KREMEN2 cells transfected for 48 h with the indicated siRNAs.
f, Violin plots showing clustered expression of KREMEN2 mRNA in SMARCB1-WT/SMARCA4-WT, SMARCB1-deficient and SMARCA4-deficient cell lines, based on an analysis of mutations, gene copy number, and gene expression data from DepMap (data version 23Q2). SMARCB1-WT/SMARCA4-WT (n = 1343), SMARCB1-deficient cell lines (n = 19), and SMARCA4-deficient cell lines (n = 37) were analyzed, and data are presented as the mean ± SEM (standard error of the mean). n indicates independent samples.
g, Box plots showing KREMEN2 mRNA signals in kidney rhabdoid tumors deficient in SMARCB1 were compared with signals in other types of kidney tumor (data obtained from a published RNA microarray data set (GSE11482). Data are presented as the mean ± SEM. Cellular mesoblastic nephromas (CMN) (n = 12), clear cell sarcomas of the kidney (CCSK) (n = 10), rhabdoid tumor (RT) (n = 10). n indicates independent samples
h, Expression of KREMEN2 pre-mRNA (relative to that in sJMU-RTK-2 -SMARCB1 cells) in JMU-RTK-2 +SMARCB1 and JMU-RTK-2 -SMARCB1 cells. Data are presented as the mean ± SD, n = 3 independent experiments.
i, Expression of KREMEN2 pre-mRNA (relative to that in JMU-RTK-2 cells) in SMARCB1-proficient (HEK293T and H460) and SMARACB1-deficient (JMU-RTK-2, G402, and NEPS) cells. Data are presented as the mean ± SD, n = 3 independent experiments.
j, Expression of KREMEN2 mRNA (relative to that in siNT-transfected cells) in SMARCB1-deficient cell lines (JMU-RTK-2, HS-ES-2R, G402, and NEPS) transfected for 48 h with the indicated siRNAs. Data are presented as the mean ± SD, n = 3 independent experiments.
k, Immunoblot analysis of KREMEN2 and β-actin expression in SMARCB1-deficient (JMU-RTK-2, HS-ES-2R, G402, and NEPS) cell lines transfected for 48 h with the indicated siRNAs. Internal KREMEN2 protein was not detected by the commercially available anti-KREMEN2 antibody; none of the bands detected by the antibody were depleted by siRNAs targeting KREMEN2, regardless of the marked knockdown of KREMEN2 mRNA by siRNA targeting KREMEN2 (see Supplementary Fig. 2j).
l, Immunoblot analysis of KREMEN2 and β-actin expression in HS-ES-2R mock and HS-ES-2R +KREMEN2 cells transfected for 48 h with the indicated siRNAs. Ectopically overexpressed KREMEN2 was detected by the commercially available anti-KREMEN2 antibody; the band was depleted by siRNA targeting KREMEN2.
m, Localization of signals generated by ATAC-seq and RNA-seq around the KREMEN2 locus in TTC1240 +SMARCB1 and TTC1240 -SMARCB1 cells (based on published ATAC-seq data and RNA-seq data; GSE124903).
n, Localization of signals generated by H3K27me3, SUZ12, H3K27ac ChIP-seq, and RNA-seq around the KREMEN2 locus in TTC1240 +SMARCB1 and TTC1240 -SMARCB1 cells (based on published ChIP-seq data and RNA-seq data; GSE90634).
o, p, Localization of signals generated by H3K4me3, H3K4me1, H3K27ac, H3K27me3, CUT&RUN-seq, p300 ChIP-seq, ATAC-seq, and RNA-seq around the ANKRD1 (o) and CDKN1A (p) loci in JMU-RTK-2 +SMARCB1 and JMU-RTK-2 -SMARCB1 cells.
For all experiments, p values were determined by an unpaired two-tailed Student' s t-test. *p < 0.05, **p < 0.01, ***p < 0.001.

Supplementary Figure 3

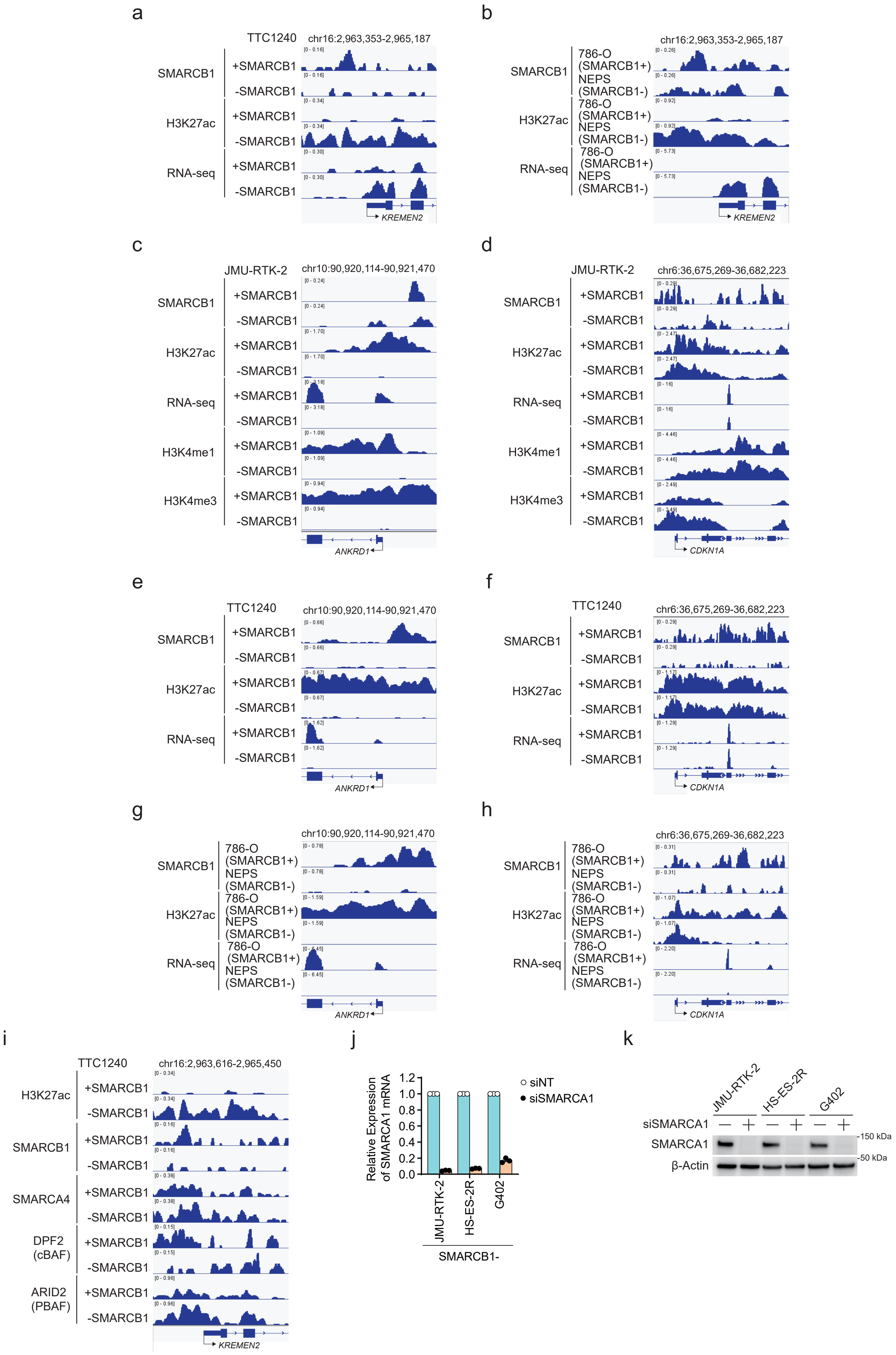

Supplementary Figure 3. SMARCB1-containg SWI/SNF complexes localize to the region upstream of the KREMEN2 locus to repress transcription.

a, Localization signals generated by SMARCB1 and H3K27ac by ChIP-seq and RNA-seq around the KREMEN2 locus in TTC1240 +SMARCB1 and TTC1240 -SMARCB1 cells (based on published ChIP-seq data and RNA-seq data; GSE124903).

b, Localization signals generated by SMARCB1 and H3K27ac by CUT&RUN-seq and RNA-seq around the KREMEN2 locus in the SMARCB1-proficient cell line 786-O and the SMARCB1-deficient cell line NEPS.

c, d, Localization signals generated by SMARCB1, H3K27ac, H3K4me1, and H3K4me3 by CUT&RUN-seq and RNA-seq around the ANKRD1 (c) and CDKN1A (d) loci in JMU-RTK-2 +SMARCB1 and JMU-RTK-2 -SMARCB1 cells.

e, f, Localization signals generated by SMARCB1 and H3K27ac ChIP-seq and RNA-seq around the ANKRD1 (e) and CDKN1A (f) loci in TTC1240 +SMARCB1 and TTC1240 -SMARCB1 cells (based on published ChIP-seq data and RNA-seq data; GSE124903).

g, h, Localization signals generated by SMARCB1 and H3K27ac CUT&RUN-seq and RNA-seq around the ANKRD1 (g) and CDKN1A (h) loci in the SMARCB1-proficient cell line 786-O and the SMARCB1-deficient cell line NEPS.

i, Localization of CUT&RUN-seq and RNA-seq signals generated by H3K27ac, SMARCB1, SMARCA4, and DPF2, which is a subunit of the cBAF complex, and ARID2, which is a subunit of the PBAF complex, around the KREMEN2 loci in TTC1240 +SMARCB1 and TTC1240 -SMARCB1 cells (based on published ChIP-seq data and RNA-seq data; GSE124903).

j, Expression of SMARCA1 mRNA (relative to that in siNT-transfected cells) in SMARCB1-deficient (JMU-RTK-2, HS-ES-2R, and G402) cell lines transfected for 48 h with the indicated siRNAs. Data are expressed as the mean ± SD, n = 3 independent experiments.

k, Immunoblot analysis of SMARCA1 and β-actin expression in SMARCB1-deficient (JMU-RTK-2, HS-ES-2R, and G402) cell lines transfected for 48 h with the indicated siRNAs.

Supplementary Figure 4

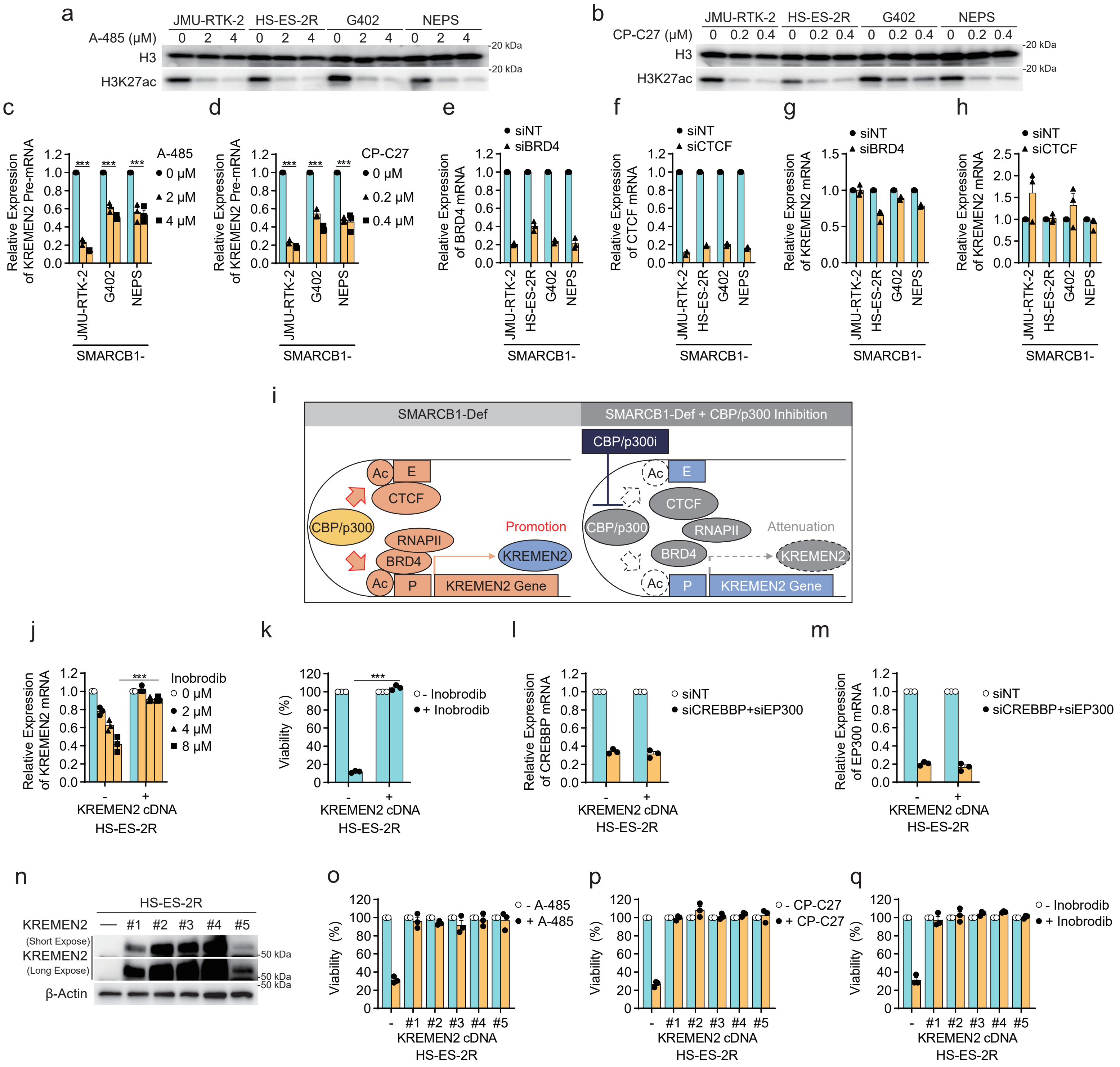

Supplementary Figure 4. Simultaneous inhibition of CBP/p300 in SMARCB1-deficient cells induces synthetic lethality by downregulating KREMEN2.

a, b, Immunoblot analysis of histone H3 and H3K27ac expression in SMARCB1-deficient cell lines (JMU-RTK-2, HS-ES-2R, G402, and NEPS) treated with the indicated concentrations of A-485 (a) and CP-C27 (b) for 24 h.

c, d, Expression of KREMEN2 pre-mRNA (relative to that in NT (non-treated) cells) in SMARCB1-deficient cell lines (JMU-RTK-2, G402, and NEPS) treated with the indicated concentrations of A-485 (c) and CP-C27 (d) for 24 h. Data are presented as the mean ± SD (standard deviation), n = 3 independent experiments.

e, f, Expression of BRD4 (e) and CTCF (f) mRNA (relative to that in siNT (non-targeting siRNA)-transfected cells) in SMARCB1-deficient cell lines (JMU-RTK-2, HS-ES-2R, G402, and NEPS) transfected with the indicated siRNAs for 48 h. Data are presented as the mean ± SD, n = 3 independent experiments.

g, h, Expression of KREMEN2 mRNA (relative to that in siNT-transfected cells) in SMARCB1-deficient cell lines (JMU-RTK-2, HS-ES-2R, G402, and NEPS) transfected with or without siRNAs targeting BRD4 (g), or CTCF (h). Data are presented as the mean ± SD, n = 3 independent experiments.

i, A schematic model showing that histone acetylation by CBP and p300 is required for recruitment of a set of transcription factors and RNAPII onto the KREMEN2 locus to promote transcription in SMARCB1-deficient cells. In SMARCB1-deficient cells, histone acetylation by CBP and p300 acts as a foundation for the transcriptional machinery at the KREMEN2 locus, and is required for recruitment of a set of transcription factors, including BRD4 and CTCF; this then facilitates transcription of the KREMEN2 gene via RNA polymerase II. In SMARCB1-deficient cells treated with a CBP/p300 inhibitor, histone acetylation reduced by CBP/p300 inhibition leads to failure to recruit transcription factors to promotor and enhancer regions at the KREMEN2 locus, resulting in attenuation of KREMEN2 gene transcription.

j, Expression of KREMEN2 mRNA (relative to that in NT cells) in HS-ES-2R mock and HS-ES-2R +KREMEN2 cells treated with the indicated concentrations of inobrodib for 24 h. Data are presented as the mean ± SD, n = 3 independent experiments.

k, Viability of HS-ES-2R mock and HS-ES-2R +KREMEN2 cells treated with 3 μM inobrodib for 6 days. Data are presented as the mean ±SEM (standard error of the mean), n = 3 independent experiments.

l, m, Expression of CREBBP (l) and EP300 (m) mRNA (relative to that in siNT-transfected cells) in HS-ES-2R mock and HS-ES-2R +KREMEN2 cells transfected with or without siRNAs targeting CREBBP+EP300 for 48 h. Data are presented as the mean ±SD, n = 3 independent experiments.

n, Immunoblot analysis of KREMEN2 and β-actin expression in clones of HS-ES-2R mock and HS-ES-2R +KREMEN2 cells.

o, p, q, Viability of the HS-ES-2R cell line and five cloned HS-ES-2R +KREMEN2 cells treated with 3 μM A-485 (o), 0.3 μM CP-C27 (p), or 3 μM inobrodib (q) for 6 days. Data are presented as the mean ± SEM, n = 3 independent experiments.

Supplementary Figure 5

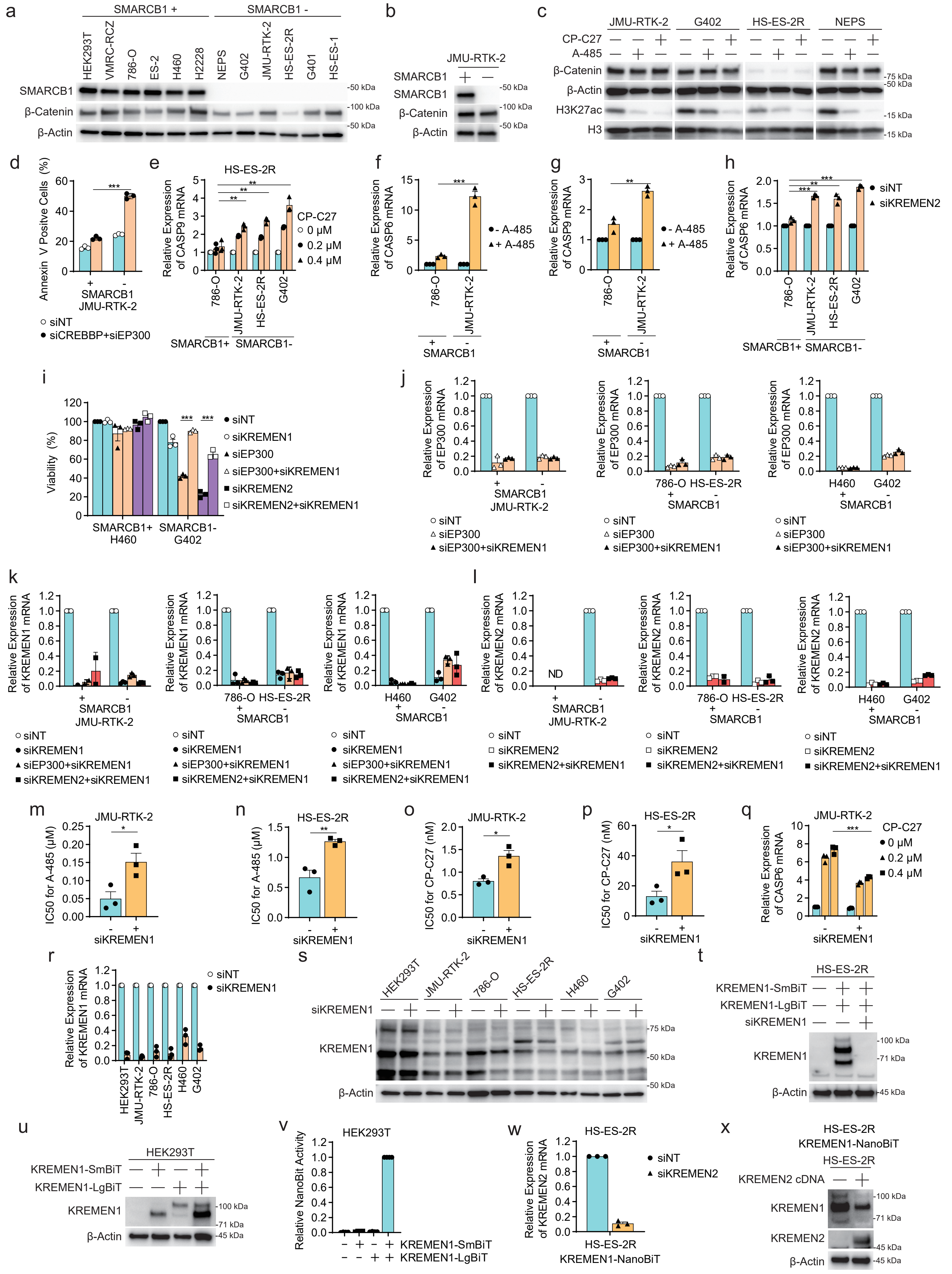

Supplementary Figure 5. Downregulation of KREMEN2 in SMARCB1-deficient cells through CBP/p300 inhibition induces apoptosis via monomerization of KREMEN1.

a, Immunoblot analysis of SMARCB1, β-catenin, and β-actin levels in SMARCB1-proficient (HEK293T, VMRC-RCZ, 786-O, ES-2, H460, and H2228) and SMARCB1-deficient (NEPS, G402, JMU-RTK-2, HS-ES-2R, G401, and HS-ES-1) cell lines.

b, Immunoblot analysis of SMARCB1, β-catenin, and β-actin levels in JMU-RTK-2 +SMARCB1 and JMU-RTK-2 -SMARCB1 cells.

c, Immunoblot analysis of β-catenin, β-actin, histone H3, and H3K27ac levels in SMARCB1-deficient (JMU-RTK-2, G402, HS-ES-2R, and NEPS) cell lines treated with 2 μM A-485 or 0.2 μM CP-C27 for 24 h.

d, Percentage of Annexin V-positive cells within JMU-RTK-2 +SMARCB1 and JMU-RTK-2 -SMARCB1 cells transfected for 96 h with or without siRNAs targeting CREBBP+EP300. Data are presented as the mean ±SD (standard deviation), n = 3.

e, Expression of CASP9 mRNA (relative to that in non-treated cells) in SMARCB1-proficient (786-O) and SMARCB1-deficient (JMU-RTK-2, HS-ES-2R, and G402) cell lines treated with 0.2 or 0.4 μM CP-C27 for 24 h. Data are presented as the mean ± SD, n = 3 independent experiments.

f, g, Expression of CASP6 (f) and CASP9 (g) mRNA (relative to that in non-treated cells) in SMARCB1-proficient (786-O) and SMARCB1-deficient (JMU-RTK-2) cell lines treated with 2 μM A-485 for 24 h. Data are presented as the mean ± SD, n = 3 independent experiments.

h, Expression of CASP6 mRNA (relative to that in siNT-transfected cells) in SMARCB1-proficient (786-O) and SMARCB1-deficient (JMU-RTK-2, HS-ES-2R, and G402) cell lines transfected for 96 h with siNT, or with siRNAs targeting KREMEN2. Data are presented as the mean ±SD, n = 3 independent experiments.

i, Viability of SMARCB1-proficient (H460) and SMARCB1-deficient (G402) cell lines transfected with siNT, or with siRNAs targeting EP300, KREMEN2, or KREMEN1. Cells were transfected with the indicated siRNAs for 48 h. The cells were then reseeded and incubated for 7 days. Data are presented as the mean ± SEM (standard error of the mean), n = 3 independent experiments.

j, Expression of EP300 mRNA (relative to that in siNT-transfected cells) in JMU-RTK-2 +SMARCB1 and JMU-RTK-2 -SMARCB1 cells, SMARCB1-proficient (786-O) and SMARCB1-deficient (HS-ES-2R) cell lines, and SMARCB1-proficient (H460) and SMARCB1-deficient (G402) cell lines transfected with siNT, or with siRNAs targeting EP300 and/or KREMEN1. Data are presented as the mean ± SD, n = 3 independent experiments.

k, Expression of KREMEN1 mRNA (relative to that in siNT-transfected cells) in JMU-RTK-2 +SMARCB1 and JMU-RTK-2 -SMARCB1 cells, SMARCB1-proficient (786-O) and SMARCB1-deficient (HS-ES-2R) cell lines, and SMARCB1-proficient (H460) and SMARCB1-deficient (G402) cell lines transfected with siNT, or with siRNAs targeting KREMEN1, EP300, or KREMEN2. Data are presented as the mean ± SD, n = 3 independent experiments.

l, Expression of KREMEN2 mRNA (relative to that in siNT-transfected cells) in JMU-RTK-2 +SMARCB1 and JMU-RTK-2 -SMARCB1 cells, SMARCB1-proficient (786-O) and SMARCB1-deficient (HS-ES-2R) cell lines, and in SMARCB1-proficient (H460) and SMARCB1-deficient (G402) cell lines, transfected with siNT, or with siRNAs targeting KREMEN2 or KREMEN1. Data are presented as the mean ± SD, n = 3 independent experiments. ND (not determined).

m, n, IC50 (50% inhibitory concentration) values for A-485 in SMARCB1-deficient cell lines (JMU-RTK-2 (m) and HS-ES-2R (n)) transfected with siNT, or with siRNAs targeting KREMEN1. Cells were transfected with the indicated siRNAs for 48 h. The cells were then reseeded and incubated for 24 h. The cells were then treated with A-485 for 6 days and IC50 values were calculated based on cell viability. Data are presented as the mean ± SEM, n = 3 independent experiments.

o, p, IC50 values for CP-C27 in SMARCB1-deficient cell lines (JMU-RTK-2 (o) and HS-ES-2R (p)) transfected with or without siRNAs targeting KREMEN1. Cells were transfected with the indicated siRNAs for 48 h. The cells were then reseeded and incubated for 24 h. The cells were then treated with CP-C27 for 6 days and IC50 values were calculated based on cell viability. Data are presented as the mean ± SEM, n = 3 independent experiments.

q, Expression of CASP6 mRNA (relative to that in non-treated cells transfected with non-targeting siRNA) in SMARCB1-deficient JMU-RTK-2 cells treated without or with CP-C27, and transfected with or without siRNA for KREMEN1. Cells were transfected with the indicated siRNAs for 48 h. The cells were reseeded and incubated for 24 h. The cells were treated without or with 0.2 or 0.4 μM CP-C27 for 24 h. Data are presented as the mean ± SEM, n = 3 independent experiments.

r, Expression of KREMEN1 mRNA (relative to that siNT-transfected cells) in SMARCB1-proficient (HEK293T, 786-O, and H460) and SMARCB1-deficient (JMU-RTK-2, HS-ES-2R, and G402) cell lines transfected with siNT, or with siRNAs targeting KREMEN1, for 48 h. Data are presented as the mean ± SD, n = 3 independent experiments.

s, Immunoblot analysis of KREMEN1 and β-actin expression in SMARCB1-proficient (HEK293T, 786-O, and H460) and SMARCB1-deficient (JMU-RTK-2, HS-ES-2R, and G402) cell lines transfected with or without siRNAs targeting KREMEN1, for 48 h. Endogenous KREMEN1 protein was not detected by the commercially available KREMEN1 antibody as none of the bands detected by the KREMEN1 antibody were depleted upon transfection of siRNA targeting KREMEN1; nevertheless, marked knockdown of KREMEN1 mRNA by siRNA targeting KREMEN1 was noted (as shown in Supplementary Fig. 5r).

t, Immunoblot analysis of KREMEN1 and β-actin expression in HS-ES-2R mock and HS-ES-2R NanoBiT cells (HS-ES-2R +KREMEN1-SmBiT +KREMEN1-LgBiT) transfected with or without siRNAs targeting KREMEN1. Ectopically overexpressed KREMEN1 proteins were detected by the commercially available KREMEN1 antibody because the bands were depleted upon transfection of siRNA targeting KREMEN1.

u, Immunoblot analysis of KREMEN1 and β-actin expression in HEK293T cells transfected for 72 h without or with KREMEN1-SmBiT and/or KREMEN1-LgBiT vectors.

v, NanoBiT activity of KREMEN1 (relative to that in HEK293T cells transfected with both KREMEN1-SmBiT and KREMEN1-LgBiT expression vectors) in HEK293T cells transfected without or with KREMEN1-SmBiT and/or KREMEN1-LgBiT vectors for 72 h. Data are presented as the mean ± SEM, n = 4 independent experiments.

w, Expression of KREMEN2 mRNA (relative to that in siNT-transfected cells) in HS-ES-2R NanoBiTcells (HS-ES-2R +KREMEN1-SmBiT +KREMEN1-LgBiT) transfected with or without siRNAs targeting KREMEN2, for 48 h. Data are presented as the mean ± SD, n = 3 independent experiments.

x, Immunoblot analysis of KREMEN1, KREMEN2, and β-actin expression in HS-ES-2R NanoBiT cells (HS-ES-2R +KREMEN1-SmBiT +KREMEN1-LgBiT) transduced without or with the KREMEN2 cDNA vector.

For all experiments, p values were determined by an unpaired two-tailed Student' s t-test. *$p < 0.05$, **$p < 0.01$, ***$p < 0.001$.

Supplementary Figure 6

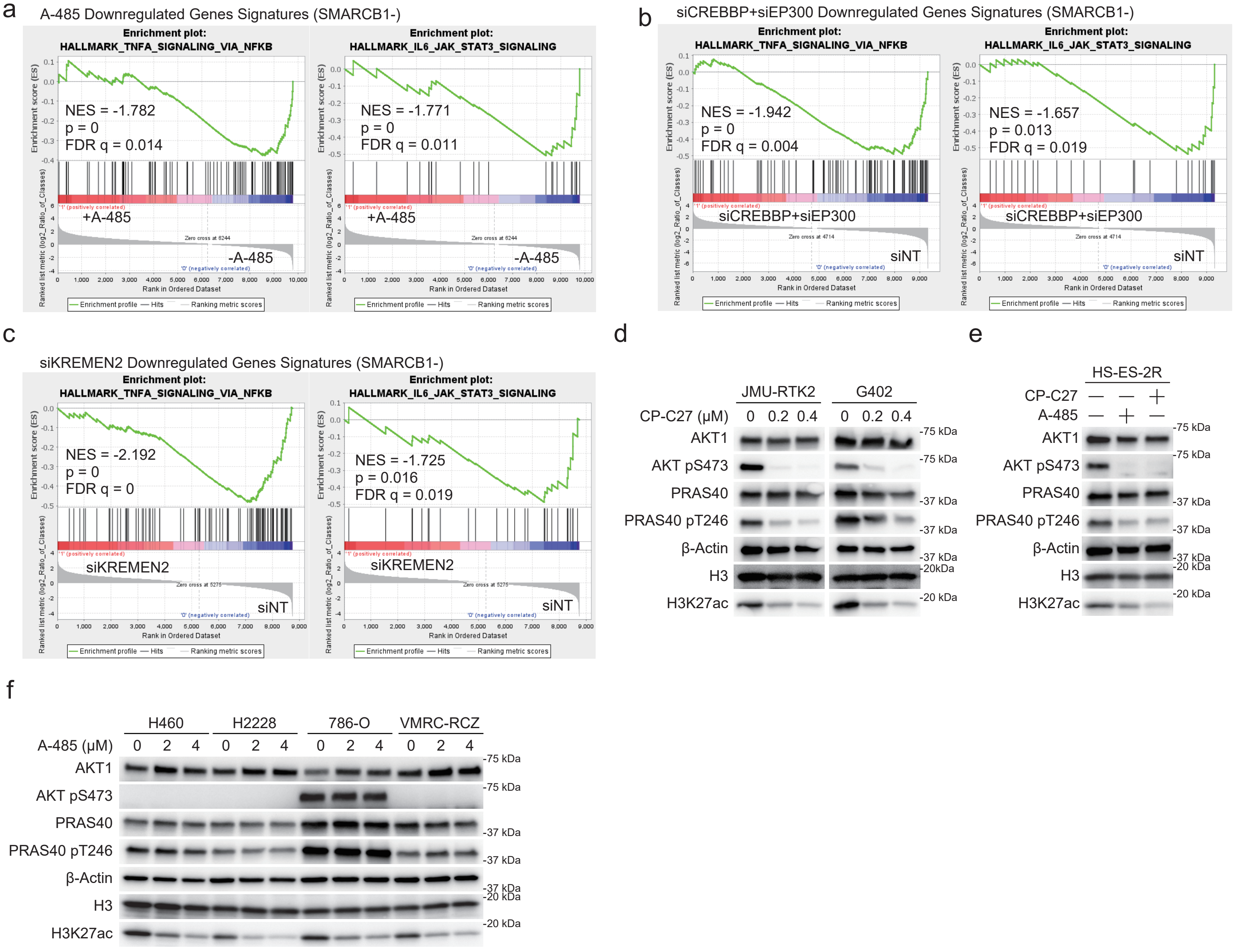

Supplementary Figure 6. Downregulation of KREMEN2 by simultaneous inhibition of CBP/p300 in SMARCB1-deficient cells suppresses anti-apoptotic signaling pathways.
a, Gene Set Enrichment Analysis (GSEA) of TNFα/NF-kB- and IL-6/JAK2/STAT3-dependent transcriptional signatures in the rank of transcripts differentially expressed between SMARCB1-deficient cell lines (JMU-RTK-2 and HS-ES-2R) treated without or with 2 μM A-485 for 96 h.
b, GSEA of TNFα/NF-kB- and IL-6/JAK2/STAT3-related signatures in the rank of transcripts differentially expressed between SMARCB1-deficient cell lines (JMU-RTK-2 and HS-ES-2R) transfected with siNT, or with siRNAs targeting CREBBP+EP300 for 96 h.
c, GSEA of TNFα/NF-kB and IL-6/JAK2/STAT3-dependent transcriptional signatures in the rank of transcripts differentially expressed between SMARCB1-deficient cell lines (JMU-RTK-2 and HS-ES-2R) transfected with siNT, or with siRNAs targeting KREMEN2 for 96 h.
d, Immunoblot analysis of AKT1, AKT pS473, PRAS40, PRAS40 pT246, histone H3, H3K27ac, and β-actin expression in SMARCB1-deficient cell lines (JMU-RTK-2, G402) treated without or with 0.2 μM or 0.4 μM CP-C27 for 16 h.
e, Immunoblot analysis of AKT1, AKT pS473, PRAS40, PRAS40 pT246, histone H3, H3K27ac, and β-actin expression in SMARCB1-deficient cell lines (HS-ES-2R) treated without or with 2 μM A-485 or 0.2 μM CP-C27 for 16 h.
f, Immunoblot analysis of AKT1, AKT pS473, PRAS40, PRAS40 pT246, histone H3, H3K27ac, and β-actin expression in SMARCB1-proficient cell lines (H460, H2228, 786-O, and VMRC-RCZ) treated without or with 2 μM or 4 μM A-485 for 16 h.

Supplementary Figure 7

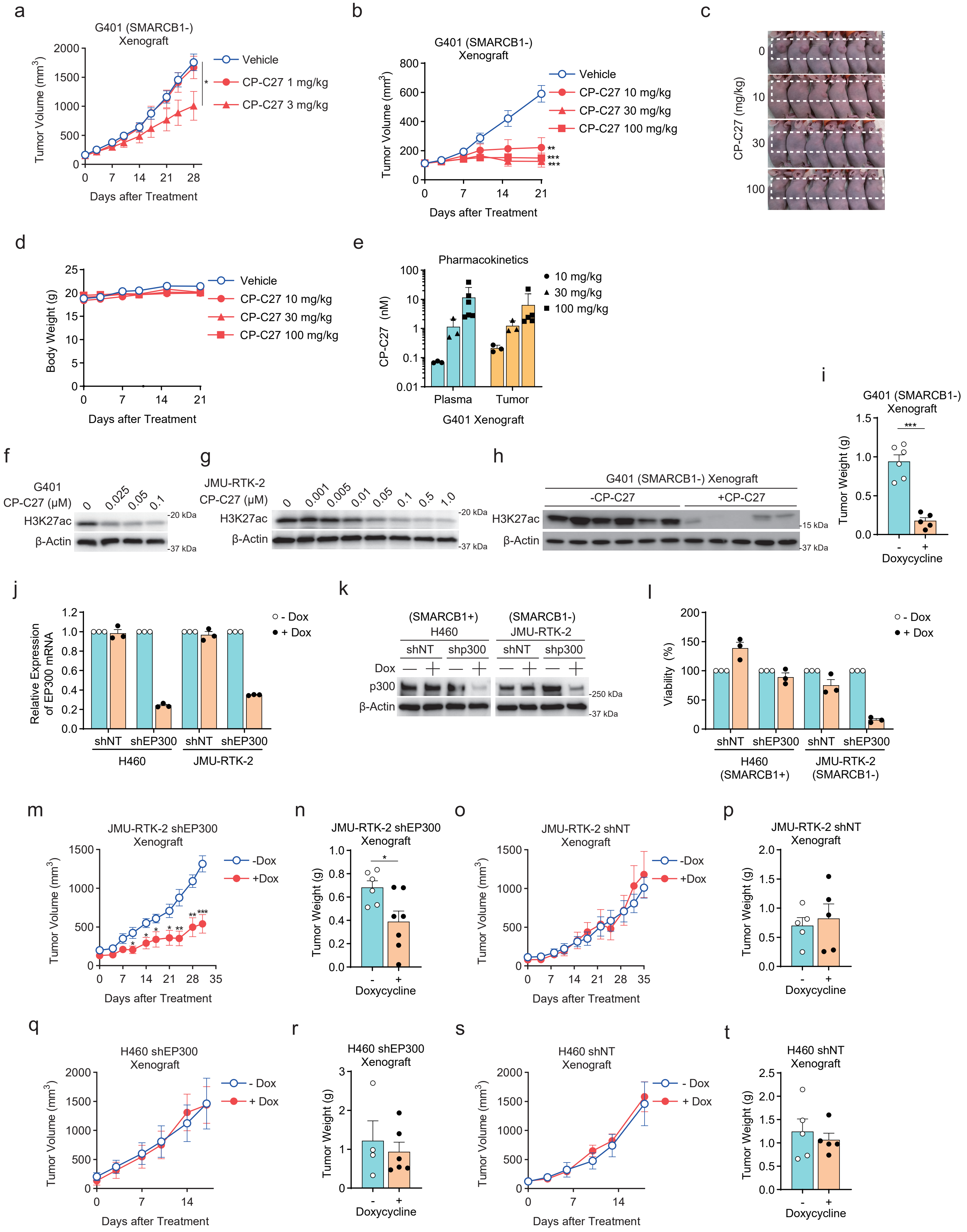

Supplementary Figure 7. Treatment with a CBP/p300 dual inhibitor suppresses tumor growth of xenografts derived from SMARCB1-deficient cancer cells.

a, Volume of xenografts derived from SMARCB1-deficient G401 cells and isolated from mice treated with CP-C27 twice a day. Data are presented as the mean ± SEM (standard error of the mean); 0 mg/kg (n = 5 biologically independent animals per group), 1 mg/kg (n = 5 biologically independent mice per group), 3 mg/kg (n = 5 biologically independent mice per group).

b, Volume of xenografts derived from SMARCB1-deficient G401 cells and isolated from mice treated with CP-C27 twice a day. Data are presented as the mean ± SEM; 0 mg/kg (n = 6 biologically independent mice per group), 10 mg/kg (n = 6 biologically independent mice per group), 30 mg/kg (n = 6 biologically independent mice per group), 100 mg/kg (n = 6 biologically independent mice per group).

c, Photographs of mice bearing tumor xenografts derived from SMARCB1-deficient G401 cells and treated with CP-C27 (described in b).

d, Body weight of mice treated with CP-C27 twice a day. Data are presented as the mean ± SEM; 0 mg/kg (n = 6 biologically independent mice per group), 10 mg/kg (n = 6 biologically independent mice per group), 30 mg/kg (n = 6 biologically independent mice per group), 100 mg/kg (n = 6 biologically independent mice per group).

e, Concentration of CP-C27 in plasma and tumor tissues obtained from mice treated with CP-C27 twice a day. Data are presented as the mean ± SEM; 10 mg/kg (n = 6 biologically independent mice per group), 30 mg/kg (n = 6 biologically independent mice per group), 100 mg/kg (n = 6 biologically independent mice per group).

f, g, Immunoblot analysis of H3K27ac and β-actin expression by SMARCB1-deficient G401 cells (f) and SMARCB1-deficient JMU-RTK-2 cells (g) treated with the indicated concentrations of CP-C27.

h, Immunoblot analysis of H3K27ac and β-actin expression in tumor xenografts derived from SMARCB1-deficient G401 cells isolated from mice treated with 10 mg/kg CP-C27 twice a day.

i, Weight of xenografts derived from SMARCB1-deficient G401 cells and isolated from mice treated with CP-C27 twice a day. Data are presented as the mean ± SEM; 0 mg/kg (n = 6 biologically independent mice per group), 10 mg/kg (n = 6 biologically independent mice per group).

j, Expression of EP300 mRNA in SMARCB1-proficient H460 shNT cells, H460 shEP300 cells, SMARCB1-deficient JMU-RTK-2 shNT cells, and JMU-RTK-2 shEP300 cells treated without or with 1 μ g/mL doxycycline (Dox) for 96 h. Data are presented as the mean ± SD (standard deviation); n = 3 independent experiments.

k, Immunoblot analysis of p300 and β-actin expression in SMARCB1-proficient H460 shNT cells, H460 shEP300 cells, SMARCB1-deficient JMU-RTK-2 shNT cells, and JMU-RTK-2 shEP300 cells treated without or with 1 μg/mL Dox for 96 h.

l, Viability of SMARCB1-proficient H460-shNT, H460-shEP300, SMARCB1-deficient JMU-RTK-2-shNT, and JMU-RTK-2-shEP300 cells treated without or with 1 μg/mL Dox. Cells were treated without or with 1 μ g/mL Dox for 48 h. The cells were then reseeded and treated without or with 1 μg/mL Dox for 48 h. The cells were then reseeded and treated without or with 1 μg/mL Dox for 6 days. Data are presented as the mean ± SEM; n = 3 independent experiments.

m, n, Volume (m) and weight (n) of xenografts derived from SMARCB1-deficient JMU-RTK-2 shEP300 cells and isolated from mice treated without or with Dox. Data are presented as the mean ± SEM; -Dox (n = 6 biologically independent mice per group), +Dox (n = 6 biologically independent mice per group).

o, p, Volume (m) and weight (n) of xenografts derived from SMARCB1-deficient JMU-RTK-2 shNT cells and isolated from mice treated without or with Dox. Data are presented as the mean ± SEM; -Dox (n = 5 biologically independent mice per group), +Dox (n = 5 biologically independent mice per group).

q, r, Volume (q) and weight (r) of xenografts derived from SMARCB1-deficient H460 shEP300 cells and isolated from mice treated without or with Dox. Data are presented as the mean ± SEM; -Dox (n = 4 biologically independent mice per group), +Dox (n = 6 biologically independent mice per group).

s, t, Volume (s) and weight (t) of xenografts derived from SMARCB1-deficient H460 shNT cells and isolated from mice treated without or with Dox. Data are presented as the mean ± SEM; -Dox (n = 5 biologically independent mice per group), +Dox (n = 5 biologically independent mice per group).

For all experiments, p values were determined by an unpaired two-tailed Student' s t-test. *p < 0.05, **p < 0.01, ***p < 0.001.

Supplementary Table 1. siRNAs used in this study.

No	Gene Symbol	Catalog Number
1	KAT2A	L-009722-02
2	KAT2B	L-005055-00
3	CREBBP	L-003477-00
4	EP300	L-003486-00
5	KAT6A	L-019849-00
6	KAT6B	L-019563-01
7	SUV39H1	L-009604-00
8	SUV39H2	L-008512-00
9	EHMT1	L-007065-02
10	EHMT2	L-006937-00
11	SETDB1	L-020070-00
12	SETDB2	L-014751-00
13	KMT2D	L-004828-00
14	KMT2C	L-007039-00
15	SMYD2	L-020291-00
16	SMYD3	L-013737-00
17	KMT5B	L-013366-01
18	KMT5C	L-018622-02
19	EZH1	L-004217-00
20	EZH2	L-004218-00
21	KDM1A	L-009223-00
22	KDM1B	L-008121-00
23	KDM2A	L-012458-02
24	KDM2B	L-014930-00
25	KDM3A	L-017301-00
26	KDM3B	L-020378-01
27	KDM6A	L-014140-01
28	KDM6B	L-023013-01
29	SMARCA1	L-011392-00
30	SMARCA5	L-011478-00
31	CHD3	L-023015-00
32	CHD4	L-009774-00
33	NSD2	L-006571-01
34	NSD3	L-012875-00
35	SETD5	L-028069-01
36	KMT2E	L-010580-00
37	PRDM16	L-007043-02
38	MECOM	L-006530-02
39	PRDM6	L-024425-03
40	PRDM12	L-013810-00
41	PRDM7	L-015181-00
42	PRDM9	L-020688-02
43	PRDM1	L-009322-00
44	PRDM11	L-020712-00
45	PRDM10	L-007029-02
46	PRDM15	L-008363-01
47	PRDM8	L-020669-00
48	PRDM13	L-007040-00
49	SETD4	L-006998-01
50	SETD6	L-014486-00
51	BAZ2A	L-020470-01
52	BAZ2B	L-020487-01
53	ATAD2	L-017603-00
54	ATAD2B	L-022640-00
55	BRPF1	L-011900-00
56	BRPF3	L-025088-01
57	BRD7	L-020297-00
58	BRD9	L-014250-02
59	BAZ1A	L-006941-00
60	BAZ1B	L-006901-00
61	BRD4	L-004937-00
62	CTCF	L-020165-00
63	KREMEN2	L-003847-00
64	KREMEN1	L-003846-00
65	EP400	L-021272-02
66	SRCAP	L-004830-00
67	INO80	L-004176-01
68	ARID1A	L-017263-00
69	ARID2	L-026945-01

Supplementary Table 2. Primers used in this study.

Distance from TSS site of the KREMEN2 gene	Primer Name	Primer Sequence (5' to 3')
KREMEN2-3kb	KREMEN2-3057-2989-FW	AGGTTGCAGTGAGCCAAGATC
KREMEN2-3kb	KREMEN2-3057-2989-RV	TTGAGACGGAGTCTTGCTCTG
KREMEN2-2kb	KREMEN2-2078-2016-FW	TGAGACTGAAGTTTGCCGCAGG
KREMEN2-2kb	KREMEN2-2078-2016-RV	AGAGGAAGTGGGTGCTCAGTG
KREMEN2-1.4kb	KREMEN2-1414-1370-FW	CTGACAGAACCTCCCTGAAC
KREMEN2-1.4kb	KREMEN2-1414-1370-RV	GGTGTCGATGGAGAAGAAAG
KREMEN2-0.8kb	KREMEN2-820-746-FW	AGTGGAGCTTCCTCCGTCAG
KREMEN2-0.8kb	KREMEN2-820-746-RV	ACACTTTCTTCGGATCCGCAC
KREMEN2-0.5kb	KREMEN2-615-553-FW	CCATTTTAAGGGCCTTCCGTC
KREMEN2-0.5kb	KREMEN2-615-553-RV	GTTCAAGTGGGAAGGTGTGG
KREMEN2-0.3kb	KREMEN2-334-256-FW	AGAGCTAGGCTCAGTGGGAG
KREMEN2-0.3kb	KREMEN2-334-256-RV	GTTCTCAGTCTCTGTCTCAG
KREMEN2-0kb	KREMEN2-26_54-FW	GACCTATCCTTGGTTGAGAG
KREMEN2-0kb	KREMEN2-26_54-RV	GGAAGAGGAGAAAGAGGAAG
KREMEN2_0.5kb	KREMEN2_345_456-FW	CCGAATGCTTCCAGGTGAATG
KREMEN2_0.5kb	KREMEN2_345_456-RV	TAGCTGTGTTGCTGCGTCTGG
KREMEN2_1kb	KREMEN2_947_1007-FW	CTGCTTTTAAGTGGGGTTGG
KREMEN2_1kb	KREMEN2_947_1007-RV	AAAGTGCTGGGATTACAGGCTG
KREMEN2_2kb	KREMEN2_1757_1834-FW	AGGAGGGTTGTTTTCGGAGTC
KREMEN2_2kb	KREMEN2_1757_1834-RV	AGTCCACAAAGCATCCCAGG
