# Supplementary material for: Targeting dependency on a paralog pair of CBP/p300 against de-repression of KREMEN2 in SMARCB1-deficient cancers
Source: Nat Commun. 2024 Jun 5;15:4770. doi: 10.1038/s41467-024-49063-w (PMC11153594; doi:10.1038/s41467-024-49063-w)
Supplement: Supplementary file 3 — Reporting Summary [file 41467_2024_49063_MOESM3_ESM.pdf]

nature portfolio

Corresponding author(s): Hideaki Ogiwara

Last updated by author(s): May 14, 2024

Reporting Summary

Nature Portfolio wishes to improve the reproducibility of the work that we publish. This form provides structure for consistency and transparency in reporting. For further information on Nature Portfolio policies, see our Editorial Policies and the Editorial Policy Checklist.

Statistics

For all statistical analyses, confirm that the following items are present in the figure legend, table legend, main text, or Methods section.

n/a	Confirmed	
☐	☒	The exact sample size (*n*) for each experimental group/condition, given as a discrete number and unit of measurement
☐	☒	A statement on whether measurements were taken from distinct samples or whether the same sample was measured repeatedly
☐	☒	The statistical test(s) used AND whether they are one- or two-sided<br>*Only common tests should be described solely by name; describe more complex techniques in the Methods section.*
☒	☐	A description of all covariates tested
☐	☒	A description of any assumptions or corrections, such as tests of normality and adjustment for multiple comparisons
☐	☒	A full description of the statistical parameters including central tendency (e.g. means) or other basic estimates (e.g. regression coefficient) AND variation (e.g. standard deviation) or associated estimates of uncertainty (e.g. confidence intervals)
☐	☒	For null hypothesis testing, the test statistic (e.g. *F*, *t*, *r*) with confidence intervals, effect sizes, degrees of freedom and *P* value noted<br>*Give P values as exact values whenever suitable.*
☒	☐	For Bayesian analysis, information on the choice of priors and Markov chain Monte Carlo settings
☒	☐	For hierarchical and complex designs, identification of the appropriate level for tests and full reporting of outcomes
☒	☐	Estimates of effect sizes (e.g. Cohen's *d*, Pearson's *r*), indicating how they were calculated

Our web collection on statistics for biologists contains articles on many of the points above.

Software and code

Policy information about availability of computer code

Data collection	No software was used.
Data analysis	HISAT2 version 2.2.1<br>Strand NGS ver 4.0<br>deepTools version 3.5.1<br>Integrative Genomics Viewer version 2.13.2<br>fastp version 0.12.4<br>Bowtie2 version 2.4.5<br>picard-tools version 2.26.11<br>Transcriptome Analysis Console version 4.0<br>GuavaSoft software (v. 2.7)<br>GraphPad Prism 8.4.3<br>Gene Set Enrichment Analysis 4.3.2

For manuscripts utilizing custom algorithms or software that are central to the research but not yet described in published literature, software must be made available to editors and reviewers. We strongly encourage code deposition in a community repository (e.g. GitHub). See the Nature Portfolio guidelines for submitting code & software for further information.

Data

Policy information about availability of data

All manuscripts must include a data availability statement. This statement should provide the following information, where applicable:

- Accession codes, unique identifiers, or web links for publicly available datasets
- A description of any restrictions on data availability
- For clinical datasets or third party data, please ensure that the statement adheres to our policy

The sequencing data sets generated during the current study are deposited in the Gene Expression Omnibus (GEO) repository. The accession numbers for the data reported in this paper are GSE237043. Human reference genome is hg38. Other data sets that were previously published and used in this study have been deposited in the Gene Expression Omnibus (GEO) repository under accession numbers GSE11482, GSE90634, GSE124903. Gene expression data from kidney-derived malignant rhabdoid tumor (MRT), cellular mesoblastic nephromas (CMN), and clear cell sarcomas of the kidney (CCSK) have been deposited in the Gene Expression Omnibus (GEO) database (GSE11482). ChIP-seq datasets for TTC1240 were obtained from publicly available NCBI GEO datasets (GSE90634, GSE124903). Mutation, copy number (CN), and gene expression datasets were obtained from the Cancer Cell Line Encyclopedia (CCLE) database and downloaded from the DepMap website (data version 23Q2, http://www.depmap.org/).

Research involving human participants, their data, or biological material

Policy information about studies with human participants or human data. See also policy information about sex, gender (identity/presentation), and sexual orientation and race, ethnicity and racism.

Reporting on sex and gender	Not applicable for this study.
Reporting on race, ethnicity, or other socially relevant groupings	Not applicable for this study.
Population characteristics	Not applicable for this study.
Recruitment	Not applicable for this study.
Ethics oversight	Not applicable for this study.

Note that full information on the approval of the study protocol must also be provided in the manuscript.

Field-specific reporting

Please select the one below that is the best fit for your research. If you are not sure, read the appropriate sections before making your selection.

☒ Life sciences ☐ Behavioural & social sciences ☐ Ecological, evolutionary & environmental sciences

For a reference copy of the document with all sections, see nature.com/documents/nr-reporting-summary-flat.pdf

Life sciences study design

All studies must disclose on these points even when the disclosure is negative.

Sample size	Sample sizes were determined based on the expected variance of the experiment and the technical limitations. Sample sizes for in vitro experiments were chosen 3 samples per each group based on the standard practices of the field. Sample sizes for in vivo experiments 4-8 tumors per each group were used to have appropriate statistical power based on previous experience.
Data exclusions	No data exclusions were performed.
Replication	For all experiments, there are at least two independent biological repeats and multiple technical repeats in each. In all instances, all the attempts at replicating the experiments produced similar results.
Randomization	Not relevant because the samples are not grouped.
Blinding	Not relevant because the samples are not grouped.

Reporting for specific materials, systems and methods

We require information from authors about some types of materials, experimental systems and methods used in many studies. Here, indicate whether each material, system or method listed is relevant to your study. If you are not sure if a list item applies to your research, read the appropriate section before selecting a response.

Materials & experimental systems

n/a	Involved in the study
☐	☒ Antibodies
☐	☒ Eukaryotic cell lines
☒	☐ Palaeontology and archaeology
☐	☒ Animals and other organisms
☒	☐ Clinical data
☒	☐ Dual use research of concern
☒	☐ Plants

Methods

n/a	Involved in the study
☐	☒ ChIP-seq
☐	☒ Flow cytometry
☒	☐ MRI-based neuroimaging

Antibodies

Antibodies used

Antibodies specific for the following proteins were used for immunoblotting:
SMARCB1 (Cell Signaling Technology, 91735), 1:1000 for immunoblotting,
CBP (Cell Signaling Technology, 7425), 1:1000 for immunoblotting,
p300 (Cell Signaling Technology, 54062), 1:1000 for immunoblotting,
H3 (Cell Signaling Technology, 4499), 1:1000 for immunoblotting,
H3K27ac (Cell Signaling Technology, 8173), 1:1000 for immunoblotting,
KREMEN2 (LSBio, LS-C165609), 1:1000 for immunoblotting,
KREMEN1 (LSBio, LS�C97716), 1:1000 for immunoblotting,
β-catenin (Cell Signaling Technology, 8480), 1:1000 for immunoblotting,
AKT1 (Cell Signaling Technology, 4691), 1:1000 for immunoblotting,
AKTpS473 (Cell Signaling Technology, 4060), 1:1000 for immunoblotting,
PRAS40 (Cell Signaling Technology, 2691), 1:1000 for immunoblotting,
PRAS40pT246 (Cell Signaling Technology, 2997), 1:1000 for immunoblotting,
SMARCA1 (Cell Signaling Technology, 12483), 1:1000 for immunoblotting,
β-actin (Cell Signaling Technology, 4970 or 5125), 1:2000 for immunoblotting,
For ChIP analysis, genomic DNA regions of interest were isolated using antibody specific for
p300 (Santa Cruz, sc-585) 4 µg for ChIP.
For CUT&RUN analysis, genomic DNA regions of interest were isolated using antibody specific for
H3K4me3 (Cell Signaling Technology, 9751), 1:100 for CUT&RUN;
H3K4me1 (Cell Signaling Technology, 5326), 1:100 for CUT&RUN;
H3K27ac (Cell Signaling Technology, 8173), 1:100 for CUT&RUN;
H3K27me3 (Cell Signaling Technology, 9733), 1:100 for CUT&RUN;
CBP (Abcam, ab253202), 1:100 for CUT&RUN;
p300 (Cell Signaling Technology, 54062), 1:100 for CUT&RUN;
EZH2 (Cell Signaling Technology, 5246), 1:100 for CUT&RUN;
SMARCB1 (Cell Signaling Technology, 91735), 1:100 for CUT&RUN;
SMARCA4 (Abcam, ab110641), 1:100 for CUT&RUN;
ARID1A (Abcam, ab182560), 1:100 for CUT&RUN;
PBRM1 (Cell Signaling Technology, 89123), 1:100 for CUT&RUN;
SS18 (Cell Signaling Technology, 21792), 1:100 for CUT&RUN;
GLTSCR1 (Cell Signaling Technology, 45441), 1:100 for CUT&RUN;
SMARCA1 (Cell Signaling Technology, 12483), 1:100 for CUT&RUN;
normal IgG (Cell Signaling Technology, 66362) 1:100 for CUT&RUN.

Validation

All antibodies were validated by the commercial vender and have been used in previous publications (example provided).
SMARCB1 (Cell Signaling Technology, 91735), Nat Commun. 2023 Dec 1;14(1):7762. doi: 10.1038/s41467-023-43498-3.
CBP for human and western blotting (Cell Signaling Technology, 7425), Nat Commun. 2023 Dec 15;14(1):8361. doi: 10.1038/s41467-023-43780-4.
p300 for human and western blotting (Cell Signaling Technology, 54062), Nat Commun. 2023 Dec 15;14(1):8361. doi: 10.1038/s41467-023-43780-4.
H3 for human and western blotting (Cell Signaling Technology, 4499), Oncol Rep. 2024 Jan;51(1):11. doi: 10.3892/or.2023.8670. Epub 2023 Nov 24.
H3K27ac for human and western blotting (Cell Signaling Technology, 8173), iScience. 2023 Nov 14;26(12):108446. doi: 10.1016/j.isci.2023.108446.
KREMEN2 for human and western blotting (LSBio, LS-C165609),
KREMEN1 for human and western blotting (LSBio, LS�C97716),
β-catenin for human and western blotting (Cell Signaling Technology, 8480), Life Sci Alliance. 2023 Nov 15;7(2):e202302247. doi: 10.26508/lsa.202302247.
AKT1 for human and western blotting (Cell Signaling Technology, 4691), Bioact Mater. 2023 Oct 14:32:277-291. doi: 10.1016/j.bioactmat.2023.10.003.
AKTpS473 for human and western blotting (Cell Signaling Technology, 4060), Oncogene. 2024 Jan;43(1):22-34. doi: 10.1038/s41388-023-02875-4.
PRAS40 for human and western blotting (Cell Signaling Technology, 2691), Oncogene. 2024 Jan;43(1):22-34. doi: 10.1038/s41388-023-02875-4.
PRAS40pT246 for human and western blotting (Cell Signaling Technology, 2997), Nat Commun. 2023 May 24;14(1):2983. doi: 10.1038/s41467-023-38740-x.
SMARCA1 for human and western blotting (Cell Signaling Technology, 12483), Nat Commun. 2021 Aug 26;12(1):5123. doi: 10.1038/s41467-021-25107-3.

β-actin for human and western blotting (Cell Signaling Technology, 4970 or 5125), J Clin Invest
. 2024 Jan 2;134(1):e163242. doi: 10.1172/JCI163242.
p300 for human and ChIP (Santa Cruz, sc-585) Oncogene. 2011 May 5;30(18):2135-46. doi: 10.1038/onc.2010.592.
H3K4me3 for human and CUT&RUN (Cell Signaling Technology, 9751), iScience. 2023 Nov 14;26(12):108446. doi: 10.1016/j.isci.2023.108446.
H3K4me1 for human and CUT&RUN(Cell Signaling Technology, 5326), J Clin Invest. 2023 Jul 3;133(13):e169993. doi: 10.1172/JCI169993.
H3K27ac for human and CUT&RUN(Cell Signaling Technology, 8173), iScience. 2023 Nov 14;26(12):108446. doi: 10.1016/j.isci.2023.108446.
H3K27me3 for human and CUT&RUN(Cell Signaling Technology, 9733), iScience. 2023 Nov 14;26(12):108446. doi: 10.1016/j.isci.2023.108446.
CBP for human and CUT&RUN (Abcam, ab253202),
p300 for human and CUT&RUN (Cell Signaling Technology, 54062), Cell Death Dis. 2023 Oct 6;14(10):653. doi: 10.1038/s41419-023-06181-5.
EZH2 for human and CUT&RUN (Cell Signaling Technology, 5246), Clin Epigenetics. 2023 Oct 19;15(1):167. doi: 10.1186/s13148-023-01583-w.
SMARCB1 for human and CUT&RUN (Cell Signaling Technology, 91735), Nat Commun. 2023 Dec 1;14(1):7762. doi: 10.1038/s41467-023-43498-3.
SMARCA4 for human and CUT&RUN (Abcam, ab110641), Nat Commun. 2022 Mar 29;13(1):1658. doi: 10.1038/s41467-022-29333-1.
ARID1A for human and CUT&RUN (Abcam, ab182560), Nat Commun. 2022 Jun 17;13(1):3501. doi: 10.1038/s41467-022-31197-4.
PBRM1 for human and ChIP (Cell Signaling Technology, 89123), Cell Rep. 2022 Oct 18;41(3):111514. doi: 10.1016/j.celrep.2022.111514.
SS18 for human and CUT&RUN (Cell Signaling Technology, 21792), Sci Adv. 2020 Jul 15;6(29):eaaz3440. doi: 10.1126/sciadv.aaz3440.
GLTSCR1 for human and CUT&RUN (Cell Signaling Technology, 45441),
SMARCA1 for human and ChIP (Cell Signaling Technology, 12483), J Exp Med. 2014 Sep 22;211(10):2119-34. doi: 10.1084/jem.20140169.
normal IgG for Rabbit and CUT&RUN (Cell Signaling Technology, 66362) J Clin Invest
. 2023 Apr 17;133(8):e167953. doi: 10.1172/JCI167953.

Eukaryotic cell lines

Policy information about cell lines and Sex and Gender in Research

Cell line source(s)	The 786-O, A-204, ES-2, HEK293T, H1048, H2009, H2228, H358, H460 cells were obtained from the American Type Culture Collection (ATCC). Caki-1, G-401, G-402, JMU-RTK-2 cells were obtained from the Japanese Collection of Research Bioresources (JCRB) Cell Bank. HS-ES-1, HS-ES-2M, HS-ES-2R cells were obtained from the Riken Cell Bank (RCB). NEPS cells were provided by Dr. Hiroyuki Kawashima.
Authentication	The 786-O, ES-2, H2228, H358, H460, G-401, G-402, JMU-RTK-2, HS-ES-1, cells were authenticated by PowerPlex 16 STR System (Promega) in 2020. Although other cell lines are not authenticated, these cell lines were used for functional experiments after less than 2 months of passaging post-receipt.
Mycoplasma contamination	All cell lines tested negative for mycoplasma.
Commonly misidentified lines (See ICLAC register)	No commonly misidentified cell lines were used in the study.

Animals and other research organisms

Policy information about studies involving animals; ARRIVE guidelines recommended for reporting animal research, and Sex and Gender in Research

Laboratory animals	5 to 6-week-old female BALB/c-nu/nu mice (CLEA or Jackson Laboratory) were used in this study. All animals were maintained at sterile conditions with an ambient temperature of 20C to 26C, humidity of 30-70% (on average 50%), and a light/dark cycle of 12 hours.
Wild animals	This study did not involve wild animals.
Reporting on sex	Xenotransplantation is difficult, as transfers to other cages with unfamiliar male mice often result in fights and unnecessary injuries. In the pilot study, female mice were used because they are cost-effective and can be easily randomized to cages.
Field-collected samples	This study did not involve samples collected from the field.
Ethics oversight	Mouse experiments were approved by the National Cancer Center (NCC) Animal Ethical Committee and the Institutional Animal Care and Use Committee of Sumitomo Pharma Co. Ltd under certificate protocol number T19-013-M06 and AN13340-B02, respectively, and were performed in accordance with the Act on Welfare and Management of Animals. The experiments were carried out according to the Standards relating to the Care and Keeping and Reducing Pain of Laboratory Animals. Mice were checked for clinical indications, tumor size and body weight as specified in the experimental licenses. Mice were killed before reaching the approved humane end points of either tumor size limit of 2,000 mm3 or body weight loss of 20% or whenever they showed apparent clinical signs of pain. The maximum tumor size/burden was never exceeded in the studies. Source data are provided for all in vivo experiments.

Note that full information on the approval of the study protocol must also be provided in the manuscript.

Plants

Seed stocks

Report on the source of all seed stocks or other plant material used. If applicable, state the seed stock centre and catalogue number. If plant specimens were collected from the field, describe the collection location, date and sampling procedures.

Novel plant genotypes

Describe the methods by which all novel plant genotypes were produced. This includes those generated by transgenic approaches, gene editing, chemical/radiation-based mutagenesis and hybridization. For transgenic lines, describe the transformation method, the number of independent lines analyzed and the generation upon which experiments were performed. For gene-edited lines, describe the editor used, the endogenous sequence targeted for editing, the targeting guide RNA sequence (if applicable) and how the editor was applied.

Authentication

Describe any authentication procedures for each seed stock used or novel genotype generated. Describe any experiments used to assess the effect of a mutation and, where applicable, how potential secondary effects (e.g. second site T-DNA insertions, mosiacism, off-target gene editing) were examined.

ChIP-seq

Data deposition

☒ Confirm that both raw and final processed data have been deposited in a public database such as GEO.

☐ Confirm that you have deposited or provided access to graph files (e.g. BED files) for the called peaks.

Data access links
May remain private before publication.

The sequencing data sets generated during the current study are deposited in the Gene Expression Omnibus (GEO) repository. The accession numbers for the data reported in this paper are GSE237043.
https://www.ncbi.nlm.nih.gov/geo/query/acc.cgi?acc=GSE237043

Files in database submission

CUT&RUN and ChIP bw files:
GSM7593481 RNA_786O_A485_2uM_24h.bw
GSM7593482 RNA_786O_NT_24h.bw
GSM7593483 RNA_G402_A485_2uM_24h.bw
GSM7593484 RNA_G402_NT_24h.bw
GSM7593485 RNA_HSES2R_A485_2uM_24h.bw
GSM7593486 RNA_HSES2R_NT_24h.bw
GSM7593487 RNA_HSES2R_siCBPsip300_96h.bw
GSM7593488 RNA_HSES2R_siKREMEN2_96h.bw
GSM7593489 RNA_HSES2R_siNT_96h.bw
GSM7593490 RNA_JMURTK2_Mock_A485_2uM_24h.bw
GSM7593491 RNA_JMURTK2_Mock_NT_24h.bw
GSM7593492 RNA_JMURTK2_Mock_siCBPsip300_96h.bw
GSM7593493 RNA_JMURTK2_Mock_siKREMEN2_96h.bw
GSM7593494 RNA_JMURTK2_Mock_siNT_96h.bw
GSM7593495 RNA_JMURTK2_SMARCB1_A485_2uM_24h.bw
GSM7593496 RNA_JMURTK2_SMARCB1_NT_24h.bw
GSM7593497 RNA_NEPS_A485_2uM_24h.bw
GSM7593498 RNA_NEPS_NT_24h.bw
GSM7593499 ATAC_JMURTK2_Mock_A2_24h.bw
GSM7593500 ATAC_JMURTK2_Mock_NT_24h.bw
GSM7593502 ATAC_JMURTK2_siCBPsip300_48h.bw
GSM7593503 ATAC_JMURTK2_siNT_48h.bw
GSM7593504 ATAC_JMURTK2_SMARCB1_NT_24h.bw
GSM7593505 BRD4_JMURTK2_Mock_A485.bw
GSM7593506 BRD4_JMURTK2_Mock_NT.bw
GSM7593507 CTCF_JMURTK2_Mock_A485.bw
GSM7593508 CTCF_JMURTK2_Mock_NT.bw
GSM7593509 H3K4me1_JMURTK2_Mock_NT.bw
GSM7593510 H3K4me1_JMURTK2_SMARCB1_NT.bw
GSM7593511 H3K4me3_JMURTK2_Mock_NT.bw
GSM7593512 H3K4me3_JMURTK2_SMARCB1_NT.bw
GSM7593513 H3K27ac_786O_NT.bw
GSM7593514 H3K27ac_JMURTK2_Mock_A485.bw
GSM7593515 H3K27ac_JMURTK2_Mock_NT.bw
GSM7593516 H3K27ac_JMURTK2_Mock_NT2.bw
GSM7593517 H3K27ac_JMURTK2_SMARCB1_NT.bw
GSM7593518 H3K27ac_NEPS_NT.bw
GSM7593519 H3K27me3_JMURTK2_Mock_NT.bw
GSM7593520 H3K27me3_JMURTK2_SMARCB1_NT.bw
GSM7593521 Input_786O_NT.bw
GSM7593522 Input_JMURTK2_Mock_NT.bw
GSM7593523 Input_JMURTK2_MockSMARCB1Mix_3.bw
GSM7593524 Input_JMURTK2_SMARCB1_NT.bw
GSM7593525 Input_NEPS_NT.bw
GSM7593526 p300_JMURTK2_Mock_1.bw

GSM7593527 p300_JMURTK2_SMARCB1_2.bw
GSM7593528 RNAPII_JMURTK2_Mock_A485.bw
GSM7593529 RNAPII_JMURTK2_Mock_NT.bw
GSM7593530 SMARCB1_786O_NT.bw
GSM7593531 SMARCB1_JMURTK2_Mock_NT.bw
GSM7593532 SMARCB1_JMURTK2_SMARCB1_NT.bw
GSM7593533 SMARCB1_NEPS_NT.bw
GSM8155704 ATAC_JMURTK2_Mock_CP2_24h.bw
GSM8155705 ATAC_JMURTK2_Mock_NT2_24h.bw
GSM8155706 BRD4_JMURTK2_Mock_CP_24h.bw
GSM8155707 CTCF_JMURTK2_Mock_CP_24h.bw
GSM8155708 H3K27ac_JMURTK2_Mock_CP_24h.bw
GSM8155709 RNA_JMURTK2_Mock_CP2_24h.bw
GSM8155710 RNA_JMURTK2_Mock_NT2_24h.bw
GSM8155711 RNAPII_JMURTK2_Mock_CP_24h.bw

Genome browser session (e.g. UCSC)

The Integrative Genomics Viewer (IGV): https://igv.org/doc/desktop/#DownloadPage/

Methodology

Replicates

All ChIP-seq and CUT&RUN data was performed in singlicate.

Sequencing depth

All ChIP-seq libraries were sequenced with ~40 million paired end reads.

Antibodies

For ChIP analysis, genomic DNA regions of interest were isolated using antibody specific for
p300 (Santa Cruz, sc-585) 4 μg for ChIP.
For CUT&RUN analysis, genomic DNA regions of interest were isolated using antibody specific for
H3K4me3 (Cell Signaling Technology, 9751), 1:100 for CUT&RUN;
H3K4me1 (Cell Signaling Technology, 5326), 1:100 for CUT&RUN;
H3K27ac (Cell Signaling Technology, 8173), 1:100 for CUT&RUN;
H3K27me3 (Cell Signaling Technology, 9733), 1:100 for CUT&RUN;
CBP (Abcam, ab253202), 1:100 for CUT&RUN;
p300 (Cell Signaling Technology, 54062), 1:100 for CUT&RUN;
EZH2 (Cell Signaling Technology, 5246), 1:100 for CUT&RUN;
SMARCB1 (Cell Signaling Technology, 91735), 1:100 for CUT&RUN;
SMARCA4 (Abcam, ab110641), 1:100 for CUT&RUN;
ARID1A (Abcam, ab182560), 1:100 for CUT&RUN;
PBRM1 (Cell Signaling Technology, 89123), 1:100 for CUT&RUN;
SS18 (Cell Signaling Technology, 21792), 1:100 for CUT&RUN;
GLTSCR1 (Cell Signaling Technology, 45441), 1:100 for CUT&RUN;
SMARCA1 (Cell Signaling Technology, 12483), 1:100 for CUT&RUN;
normal IgG (Cell Signaling Technology, 66362) 1:100 for CUT&RUN.

Peak calling parameters

Raw sequencing data from ChIP-seq, CUT&RUN-seq, and ATAC-seq were trimmed using fastp version 0.12.4 35 and mapped to the human reference genome (hg38) using Bowtie2 version 2.4.5, with parameters -k 1 --no-mixed --no-discordant -X 2000. Prior to all downstream analyses, duplicate reads were removed using the MarkDuplicates command in picard-tools version 2.26.11 (http://broadinstitute.github.io/picard). From the ChIP-seq and CUT&RUN data, CPM values in the genome tracks were calculated by subtracting those in the input tracks as the background value for each cell. BigWig files were generated using the bamCompare command from deepTools version 3.5.1, with parameters: --operation subtract --normalizeUsing CPM --scaleFactorsMethod None --binSize 10 --smoothLength 30 and visualized by the Integrative Genomics Viewer version 2.13.2.

Data quality

CUT&RUN-seq and ChIP-seq tend to generate non-specific background signals. Therefore, we added snapshots of ChIP-seq and CUT&RUN-seq signals at the ANKRD1 and CDKN1A regions as “positive regions” in which SMARCB1 binding to loci outside the KREMEN2 locus has been reported. The localization signals of H3K4me3, H3K4me1, H3K27ac, p300 ChIP-seq in addition to ATAC-seq and RNA-seq were detected in the ANKRD1 gene region in JMU-RTK-2 +SMARCB1 cells, whereas that of H3K27me3 was detected in JMU-RTK-2 -SMARCB1 cells. The localization signals of H3K4me3, H3K4me1, H3K27ac, p300 ChIP-seq, ATAC-seq, and RNA-seq were detected in the CDKN1A region in both JMU-RTK-2 +SMARCB1 cells and JMU-RTK-2 -SMARCB1 cells, but that of H3K27me3 ChIP-seq was not detected in either JMU-RTK-2 +SMARCB1 or JMU-RTK-2 -SMARCB1. These snapshots support the data showing that the histone markers and p300 localize at the KREMEN2 locus. In addition, we incorporated positive control snapshots of the ANKRD1 and CDKN1A loci, both of which recruit the SMARCB1-containing SWI/SNF complex. At both the ANKRD1 and CDKN1A gene loci, SMARCB1 co-localized with H3K27ac and H3K4me3 at regions upstream of the TSS in JMU-RTK-2 +SMARCB1 cells, but not JMU-RTK-2 -SMARCB1 cells (Supplementary Fig. 3c, d). Similar results were obtained for another SMARCB1 isogenic (TTC1240) cell line model (Supplementary Fig. 3e, f) 27,35,36, and for a pair of SMARCB1-proficient 786-O cell lines and SMARCB1-deficient NEPS cells (Supplementary Fig. 3g, h). These snapshots support the data showing that the SMARCB1 localizes at the KREMEN2 locus. Localization of H3K4me3, H3K4me1, H3K27ac, H3K27me3, p300, SMARCB1 to KREMEN2 regions was also confirmed by CUT&RUN-qPCR. It can be argued therefore that the data regarding localization to KREMEN2 are reliable.

Software

fastp version 0.12.4
Bowtie2 version 2.4.5
MarkDuplicates command in picard-tools version 2.26.11
bamCoverage command from deepTools version 3.5.1

Flow Cytometry

Plots

Confirm that:

☒ The axis labels state the marker and fluorochrome used (e.g. CD4-FITC).

☒ The axis scales are clearly visible. Include numbers along axes only for bottom left plot of group (a 'group' is an analysis of identical markers).

☒ All plots are contour plots with outliers or pseudocolor plots.

☒ A numerical value for number of cells or percentage (with statistics) is provided.

Methodology

Sample preparation	The Annexin V–FITC/PI Apoptosis Detection Kit (Roche, 11858777001) was used to detect apoptotic cells. The cell pellet was suspended in 1× binding buffer and then incubated with Annexin V–FITC and PI in the dark for 10 min at 25°C.
Instrument	Fluorescence was analyzed on a Guava easyCyte HT (Millipore).
Software	The percentage of Annexin V-positive cells was calculated using GuavaSoft software (v. 2.7).
Cell population abundance	Annexin V–FITC positive fractions were detected by fractions with higher FITC signal intensity compared to abundant fractions in non-treatment samples.
Gating strategy	Live cells were gated based on SCC and FSC area parameters.

☒ Tick this box to confirm that a figure exemplifying the gating strategy is provided in the Supplementary Information.
